# Frost resistance investigation of fiber reinforced recycled brick aggregate cementitious materials

**DOI:** 10.1038/s41598-022-19006-w

**Published:** 2022-09-12

**Authors:** Yongcheng Ji, Hongrui Zhang

**Affiliations:** College of Civil Engineering, Northeast Forestry University, Harbin, 150040 China

**Keywords:** Civil engineering, Sustainability

## Abstract

In order to solve the problem of environmental pollution caused by construction waste, one typical waste of red bricks was selected as raw material in recycled concrete. This study presented recycled concrete by substituting some natural aggregates with treated red brick aggregates to study and analyze the degradation law mechanism of recycled brick aggregates concrete in the cold region. A total of fifteen categories of specimens and three experimental parameters were considered, which included numbers of freeze–thaw cycles (0, 50, and 100), steel fiber admixtures (0, 1, and 2%), and brick aggregate substitution rates (0, 25, 50, 75, and 100%), respectively. The quick freeze–thaw test method was selected to investigate recycled concrete's degradation mass loss rate and relative dynamic elastic modulus under various freeze–thaw cycles. The digital microscope and SEM were used to observe the internal microstructural changes in the specimens under different freeze–thaw times. In addition, the specimens’ microscopic damage morphology and damage mechanism were analyzed. Finally, the flexural strength of the frost-damaged specimens was tested to analyze the mechanical deterioration of the recycled concrete, and the numerical model corresponding to steel fiber dosing and recycled aggregate replacement rate was presented. The gray correlation analysis was used to quantify the influence of each experimental variable on the corresponding experimental indexes under various freeze–thaw cycles. Results showed that the specimen's mass decreased after freeze–thaw cycles, and the highest mass loss was found for the specimens with 50 and 75% brick substitution rates. In addition, the specimens showed the best relative dynamic modulus and the maximum flexural strength when the steel fiber doping was 1%. The numerical model agreed with experimental data and effectively predicted the specimens' mass loss rate, relative dynamic modulus, and flexural strength after freeze–thaw cycles. The gray correlation analysis showed that the steel fiber contents had a maximum correlation with the flexural strength, the brick substitution rates for the relative dynamic modulus, and mass loss controls the freeze–thaw cycles.

## Introduction

With the gradual acceleration of urbanization in China, many construction wastes, mainly waste concrete and red bricks, are generated every year. Nowadays, China’s annual construction waste has reached more than 100 million tons, which has accounted for 30–40% of the total urban waste^1^. The existence of construction waste not only occupies a large number of land resources but also causes severe pollution to the environment. Thus, much attention focuses on concrete recycling. However, the recycled aggregate has poorer physical properties than natural aggregate in recycled concrete, making recycled concrete's mechanical properties and durability, especially freeze–thaw resistance, weaker than ordinary concrete. All these observations have seriously hindered recycled concrete promotion in the cold regions.

In order to improve the freeze–thaw resistance of recycled concrete, scholars at home and abroad have conducted extensive research on this subject, and some scholars have used the recycled aggregate replacement rate and the number of freeze–thaw cycles as test parameters^2,3^. Fundamental studies were carried out by experimental methods such as fast-freeze and slow-freeze methods, which led to the derivation of computational models for recycled aggregate concrete under different substitution rates and different numbers of freeze–thaw cycles^4,5^. That is used to analyze the freeze–thaw damage pattern and optimal replacement rate of recycled concrete. Xiao et al.^6^ studied recycled concrete's physical and mechanical properties under the coupling effect of freeze–thaw cycles and sulfate environment with different aggregate replacement rates by formulating recycled concrete with different replacement rates and determining the optimum admixture of recycled aggregates with the help of model analysis. Su et al.^7^ analyzed the bond behavior of recycled coarse aggregate concrete concerning the number of salt frost cycles, and a prediction model for the bond-slip of RAC was established. The effect of salt frost cycles on the bond stress with reinforcement distribution was also investigated. Hao et al.^8^ reinforced Class II and III recycled concrete by adding mineral admixtures and polypropylene fibers and selected the optimal frost resistance category. The results show that the exponential model of cumulative damage D = aebN is obtained by fitting the experimental results of the freeze–thaw damage degree nonlinearly, and the model fitting equation showed high accuracy.

Due to the limitations of the recycled aggregate itself, some scholars studied the experiments by changing the admixture dose or modifying the treatment of recycled aggregate after the initial understanding of the frost resistance of recycled concrete^9,10^. The test of compressive strength, relative dynamic modulus, and other indices were used to improve the frost resistance of recycled aggregate concrete comprehensively, and the results were mostly fitted with experimental data to justify the prediction of the optimum admixture or proportion^11,12^. Peng et al.^13^ studied the variation of mechanical and durability performance of recycled concrete with different polypropylene fiber admixtures and the freeze–thaw effect. The optimum fiber admixture for the freeze–thaw cycle condition was obtained. Kazmi et al.^14^ showed that the concrete durability performance could be estimated from the physical properties of aggregates by performing freeze–thaw and sulfate attack resistance tests, and a regression model was developed considering recycled concrete specimens treated in different acidic environments. Lu et al.^15^ studied the freeze–thaw resistance of recycled concrete with different replacement rates under simulated acid rain spraying. The results showed that the freeze–thaw resistance of recycled concrete with different replacement rates was poorer than that of ordinary concrete. The acid rain attack had no significant effect on the mass loss rate of recycled concrete but significantly affected the relative dynamic elastic modulus.

Although the above studies focus on the frost resistance of recycled concrete, the test results have some limitations due to the significant differences between their test materials, test environment, test methods, and the actual situation. In addition, some studies began to wrap high-performance materials, such as carbon fiber, and combine with recycled aggregate replacement rate and several freeze–thaw cycles to consider the frost resistance and mechanical properties of recycled aggregate concrete in a comprehensive way^16–19^. For example, Liu et al.^20^ used three computational software methods, artificial neural network (ANN), Gaussian process regression (GPR), and multiple adaptive regression splines (MARS), to simulate the frost durability of recycled concrete. He et al.^21^ systematically investigated the effects of recycled coarse aggregate replacement rate and the sequence of freeze–thaw carbon fiber fabric (CFRP) reinforcement on the bearing capacity of recycled concrete short column specimens under the action of freeze–thaw cycles. An ultimate bearing capacity equation was presented to simulate recycled concrete short column specimens under the joint influence of freeze–thaw cycles and CFRP reinforcement. Zheng^22^ discussed the effect of nano-silica compounded with basalt fibers in recycled concrete on mechanical properties and durability performance and summarized the possible applications of nanomaterials, fibers, and fiber-nanomaterial modified recycled concrete in the construction industry. Liu^23^ et al. investigated an integrated machine learning-based method for predicting the sulfate root resistance of RAC systems. Four integrated learning methods, random forest, adaptive augmentation, gradient augmentation, and extreme value gradient augmentation, were used to build the prediction model. Ten variables related to material properties and environmental conditions were selected as inputs. The results show that the environmental conditions influence the sulfate resistance of recycled concrete in dry conditions. Liu^24^ et al. evaluated the use of denitrifying bacteria as a solution for treating recycled concrete. It was observed that both biologically induced calcium carbonate filled the transition zone between the old and new interfaces by thermogravimetric analysis and scanning electron microscopy. The bacteria could strengthen the adhesion between the agglomerates and the matrix and enhance the resistance to freeze–thaw cyclic stress.

The research mentioned above on recycled concrete mainly focuses on recycled concrete aggregates, while few types of research focus on recycled concrete from red brick aggregates. The red waste bricks are mainly disposed of by simple piling and landfill, which occupy land resources and pollute the environment. The physical and mechanical properties of red brick aggregate are poor. Steel fiber has high tensile strength and strong toughness, and adding steel fiber to recycled concrete can effectively improve the defects of recycled aggregate, such as low strength and many micro-cracks. Based on the better physical properties and economic applicability of steel fibers, the incorporation of steel fibers can effectively alleviate the lower mechanical properties of recycled aggregate concrete due to the limitation of fundamental physical properties of recycled aggregate. Therefore, end-hooked steel fibers were considered as additives in this study. The mass replacement rate of recycled brick aggregate, steel fiber content, and several freeze–thaw cycles were used to investigate the durability of recycled brick aggregate concrete. In addition, the mass loss, relative dynamic modulus, and flexural strength test are selected to quantify the deterioration of recycled concrete after the variation of freeze–thaw cycles. Furthermore, the analysis establishes the calculation model of brick aggregate recycled concrete after being subjected to freeze–thaw cycles. Finally, it explores the influence of each test variable on the test index of brick aggregate recycled concrete under different test environments.

## Test overview

### Test materials

**Figure 1:**
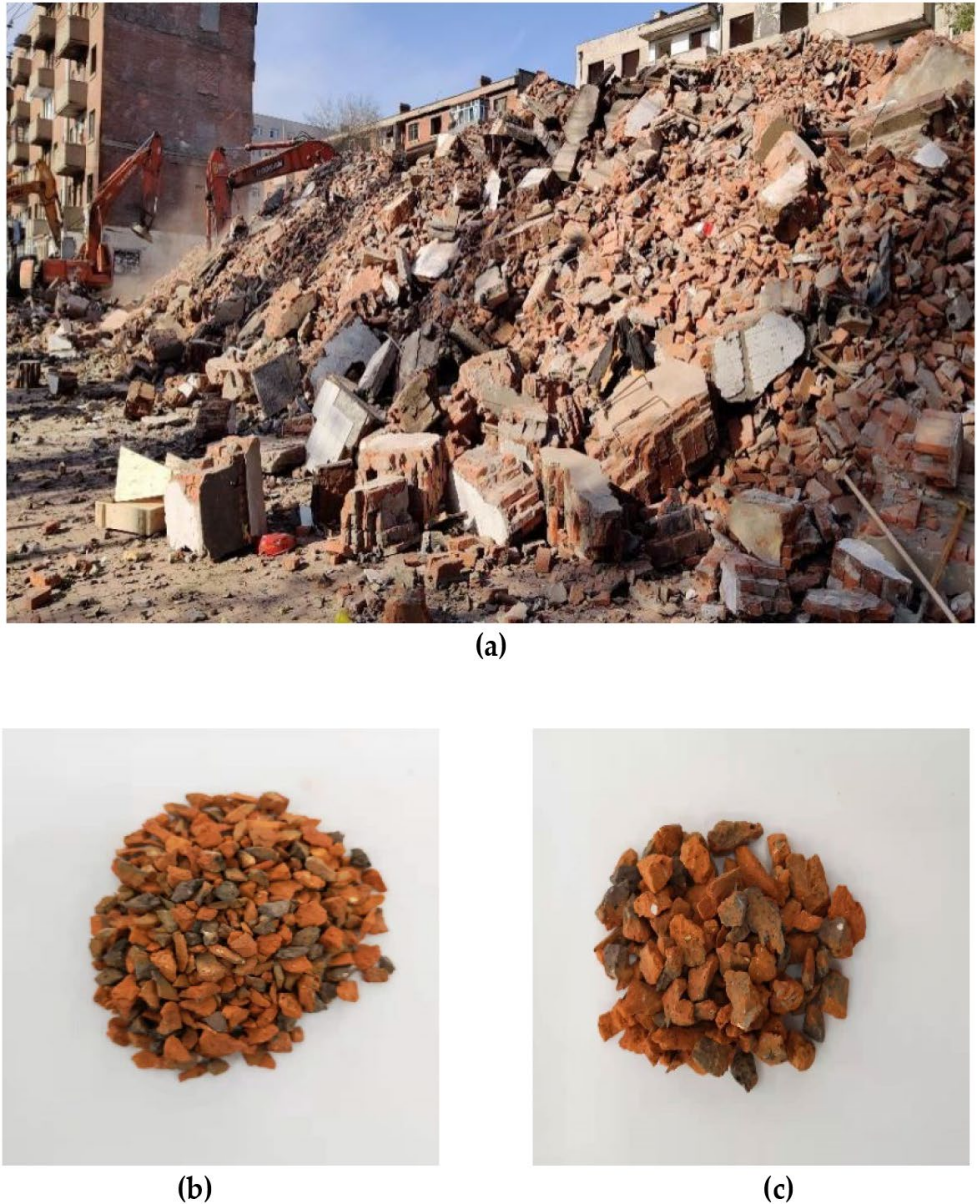
Red brick aggregate: (a) Red brick fetching site; (b) 5 ~ 10 mm particle size; (c) 10 ~ 20 mm particle size.

The recycled brick concrete ingredient comprises red brick aggregate, natural coarse aggregate, natural river sand, ordinary Portland cement, end hook steel fiber, and water. The relevant material characteristics are listed as follows:

Coarse aggregate: The recycled brick aggregate was obtained by random sampling in Xiangfang District, Harbin City (Fig. 1a). The red brick aggregate after crushing and processing by jaw crusher was used processed from the demolished bricks shown in Fig. 1. According to the ‘Standard for Quality and Inspection Methods of Sand and Stone for Ordinary Concrete (JGJ52-2006)’, the coarse aggregates of 5–10 mm (Fig. 1b) grain size and 10–20 mm (Fig. 1c) grain size were compounded in a mass ratio of 7:3 to meet the requirements of 5–20 mm continuous gradation^25^. The performance of natural coarse aggregate (NA) meets the requirements of the indicators in ‘Pebbles and Crushed Stone for Construction (GB/T14685-2011)’^26^. The main performance indexes of natural coarse aggregate (NA) and red brick recycled coarse aggregate (CCB) are shown in Table 1.

**Table 1:** Coarse aggregate performance index.

Aggregate type	Packing density (kg/m^3^)	Apparent density (kg/m^3^)	Water absorption (%)	Crush index (%)	Mud content (%)	Needle flake content (%)
NA	1425	2710	0.7	10.5	0.4	5.8
CCB	1210	2590	1.4	31.8	0.8	7.9

Cement: P.O 42.5 grade ordinary Portland cement was used for the cement, and its performance meets the requirements of the 'General Silicate Cement (GB175-2007)'^27^.

Sand: medium sand with a fineness modulus of 2.4 was used after filtering and drying.

**Figure 2:**
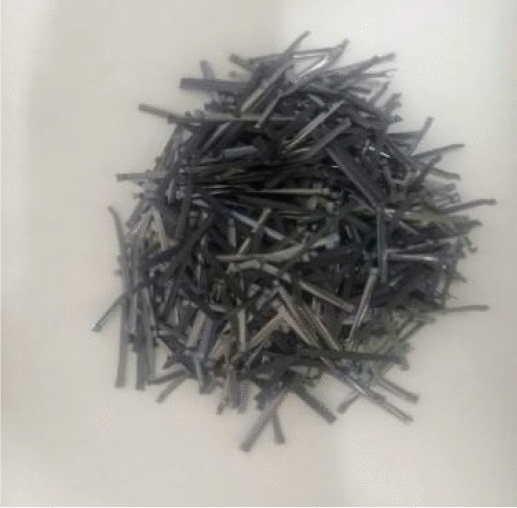
End hook type steel fiber.

Steel fiber: the end hook type of steel fiber was used, with a length of 32.0 mm ± 2.0 mm and width of 2.6 mm ± 1.2 mm. The tensile strength is ≥ 700 MPa, and the density is 7850 kg/m3, as shown in Fig. 2.

### Specimen shape and mix ratio

The specimens are grouped by the number of freeze–thaw cycles (0, 50, and 100 times), considering the steel fiber dosing and recycled aggregate replacement rate as the test parameters. Forty-five prismatic specimens were tested for mass loss, relative dynamic modulus loss, and flexural strength when subjected to freeze–thaw cycles. In addition, the deterioration mechanism of recycled concrete was discussed with various freeze–thaw processes.

The design strength of the control concrete specimen is 40 MPa in this test. Recycled concrete specimens were prepared for each group at 0, 1, and 2% of steel fiber admixture and at 0, 25, 50, 75, and 100% mass replacement of recycled coarse aggregate, respectively. Table 2 shows the concrete mix design, and NAC indicates ordinary concrete specimen, RBAC indicates recycled brick aggregate concrete specimen, "25, 50, 75, 100" represents red brick content of 25, 50, 75, 100%, "S0, S1, S2" represents steel fiber content of 1, 2, 3%, respectively. For example, "RBAC-25-S0" indicates that the red brick content is 25%, and the steel fiber admixture is 0% of the recycled brick aggregate specimen.

**Table 2:** Concrete mix design (kg/m^3^).

Concrete group	Cement	Water	Sand	NA/CCB	Steel fiber
NAC-S0	485	196	620	1138/0	0
RBAC-25-S0	485	196	620	284.5/853.5	0
RBAC-50-S0	485	196	620	569/569	0
RBAC-75-S0	485	196	620	853.5/284.5	0
RBAC-100-S0	485	196	620	0/1138	0
NAC-S1	485	196	620	1138/0	78.5
RBAC-25-S1	485	196	620	284.5/853.5	78.5
RBAC-50-S1	485	196	620	569/569	78.5
RBAC-75-S1	485	196	620	853.5/284.5	78.5
RBAC-100-S1	485	196	620	0/1138	78.5
NAC-S2	485	196	620	1138/0	157
RBAC-25-S2	485	196	620	284.5/853.5	157
RBAC-50-S2	485	196	620	569/569	157
RBAC-75-S2	485	196	620	853.5/284.5	157
RBAC-100-S2	485	196	620	0/1138	157

Considering the characteristics of high water absorption and crushing index, the coarse aggregate of recycled red brick had a saturated surface dry treatment. Therefore, the crushed and sieved recycled red brick coarse aggregate was placed in water and soaked for 24 h. Subsequently, the recycled aggregate was taken out and dried for 2 h. After treatment, it was considered to have reached a saturated surface dry state^28^.

### Test preparation and experimental method

**Figure 3:**
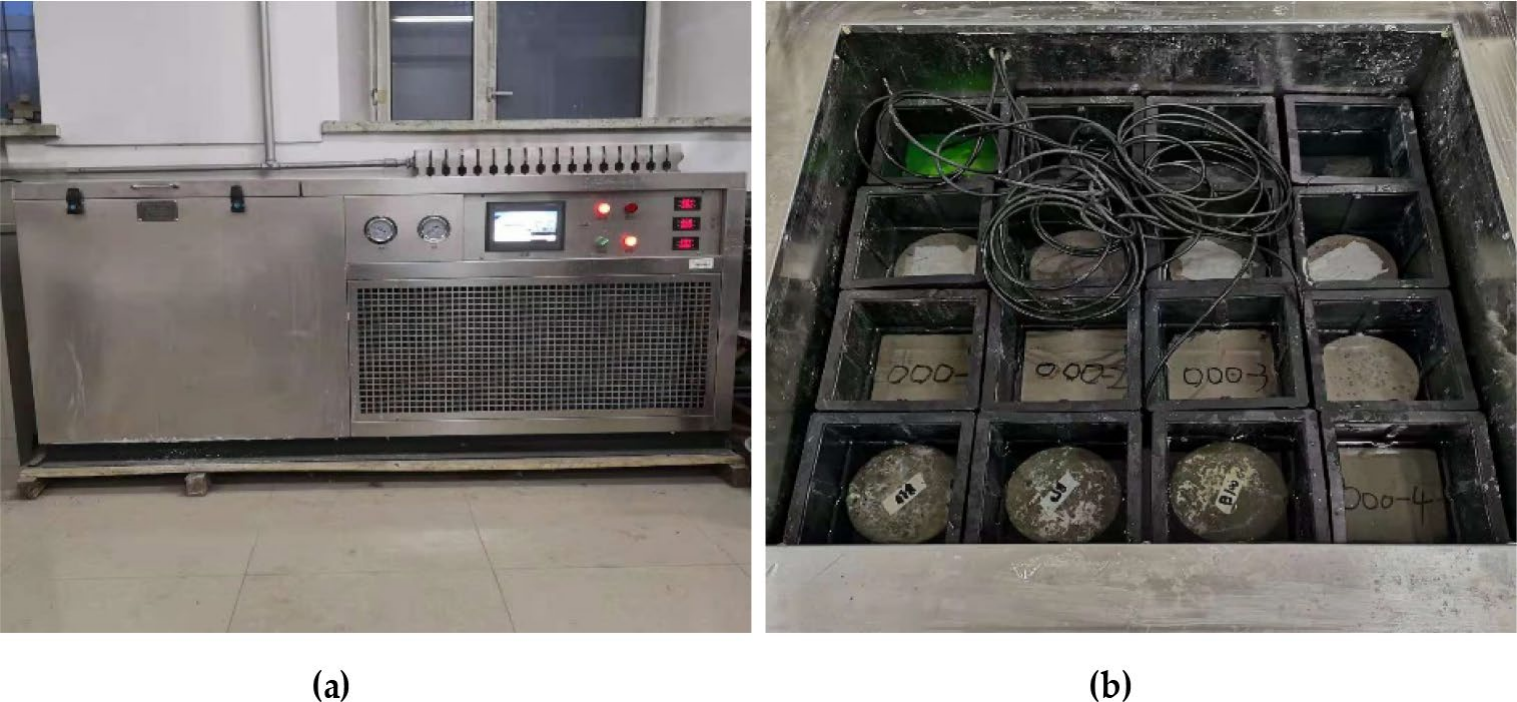
Freeze thaw circulation box: (**a**) Freeze–thaw cycle box exterior; (**b**) Inside of freeze–thaw circulation box.

**Figure 4:**
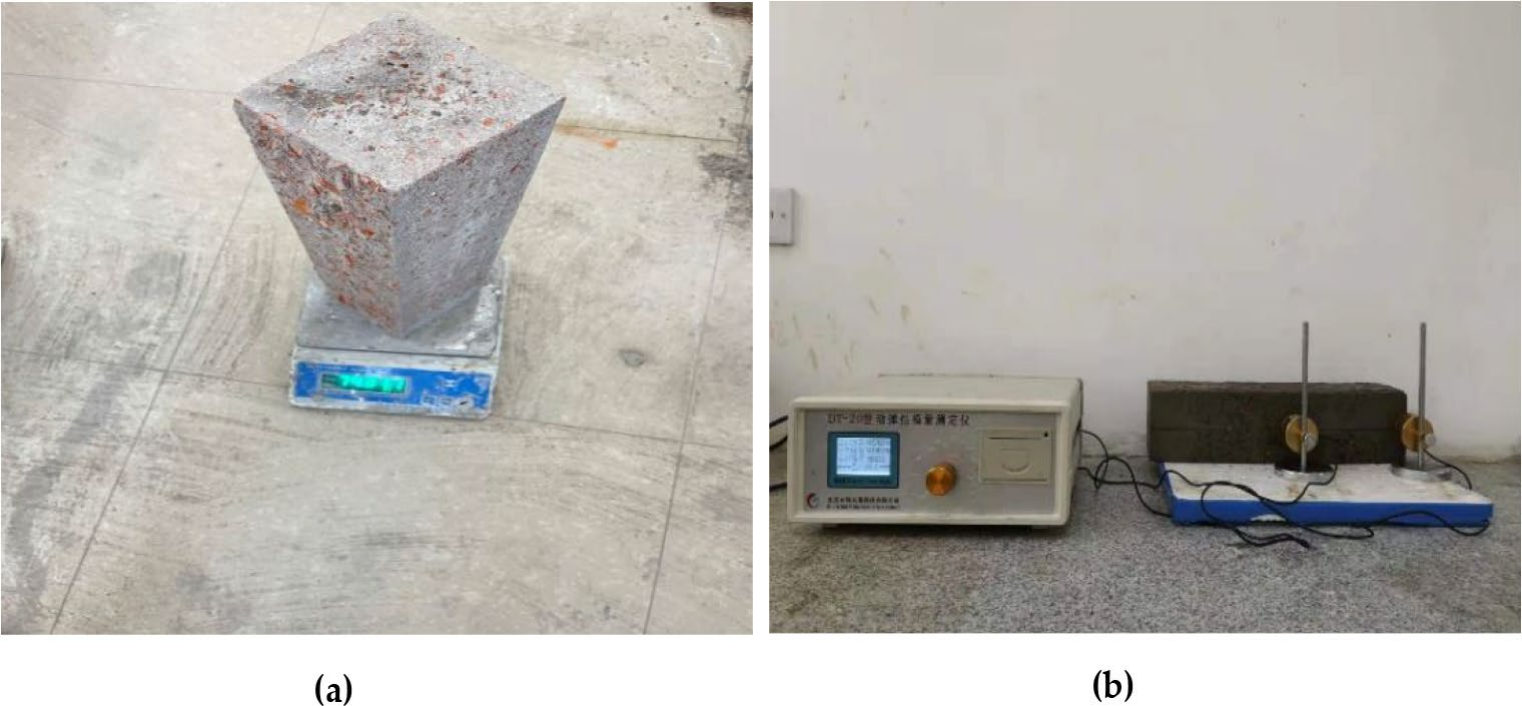
Mass loss rate and modulus of elasticity test: (**a**) Quality loss test; (**b**) Modulus of elasticity test.

**Figure 5:**
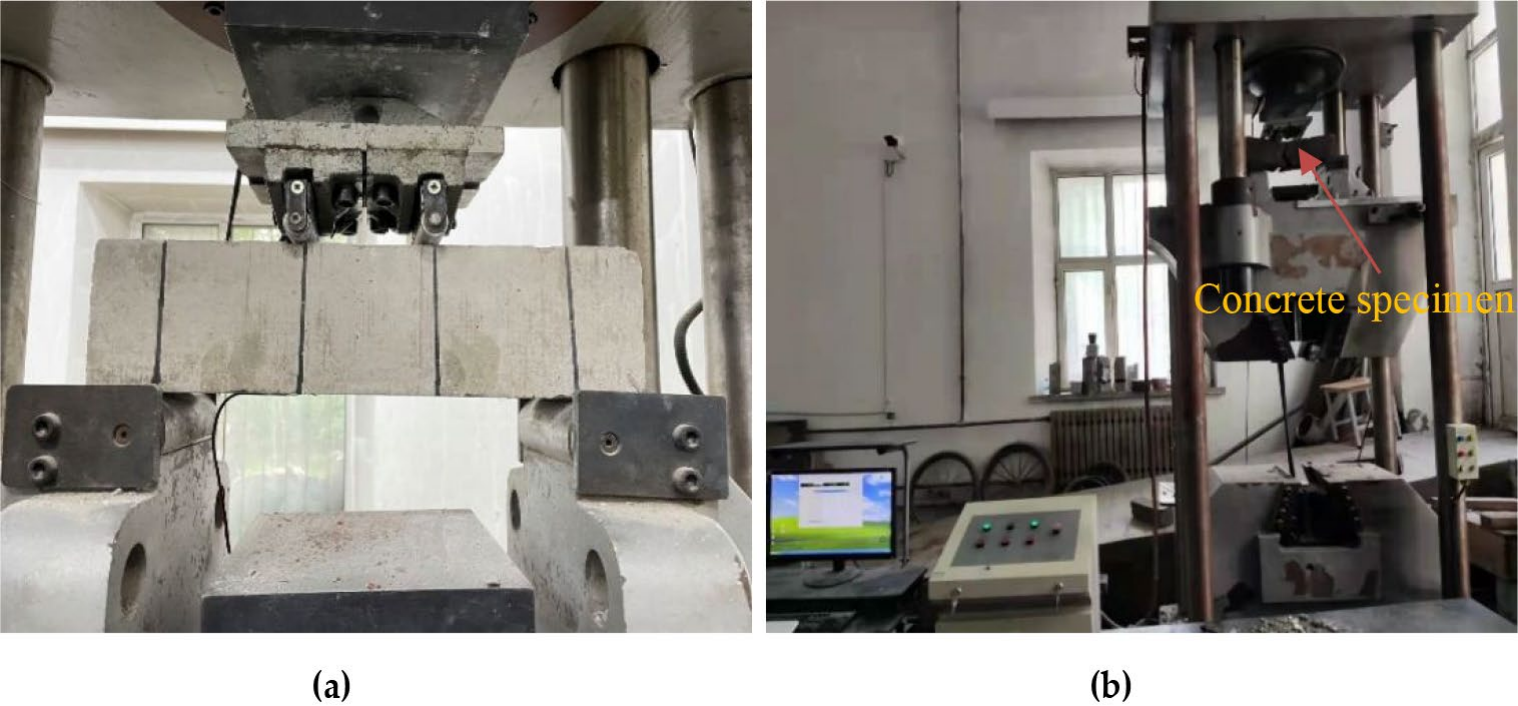
Prismatic specimen flexural test: (**a**) Prismatic flexural strength test (close up); (**b**) Prismatic flexural strength test (Vision).

According to the 'Standard for Test Methods for the Performance of Ordinary Concrete Mixes (GB/T20081-2002)', a forced mixer was used to mix the coarse and fine aggregates, then added cement gradually adding water, and finally scattered adding steel fibers^29^. The specimens were placed in a freeze–thaw cycle chamber (Fig. 3) after standard maintenance conditions for 28d. The mass loss (Fig. 4a) and relative dynamic modulus (Fig. 4b) were measured once every 25 freeze–thaw cycles, and the flexural strength of each group of specimens was tested on a compression-testing machine (Fig. 5). In addition, the ability of concrete specimens to resist bending fracture under different dosing and freeze–thaw times were tested, and the frost resistance was analyzed. Each prismatic specimens category used three samples, taking the average value as the test value to keep the data consistency. According to the 'Standard for Physical and Mechanical Properties of Concrete Test Methods (GB/T 50081-2019)', the dimension of the flexural test specimen is 100 mm in height by 100 mm in width by 400mmin length^30^. The freeze–thaw test was conducted in a freeze–thaw cycle chamber and was referred to as 'Standard for long-term performance and durability test methods for ordinary concrete (GB/T50082-2009)'^31^.

## Specimen damage morphology and mechanism analysis

### Analysis of damage patterns

**Figure 6:**
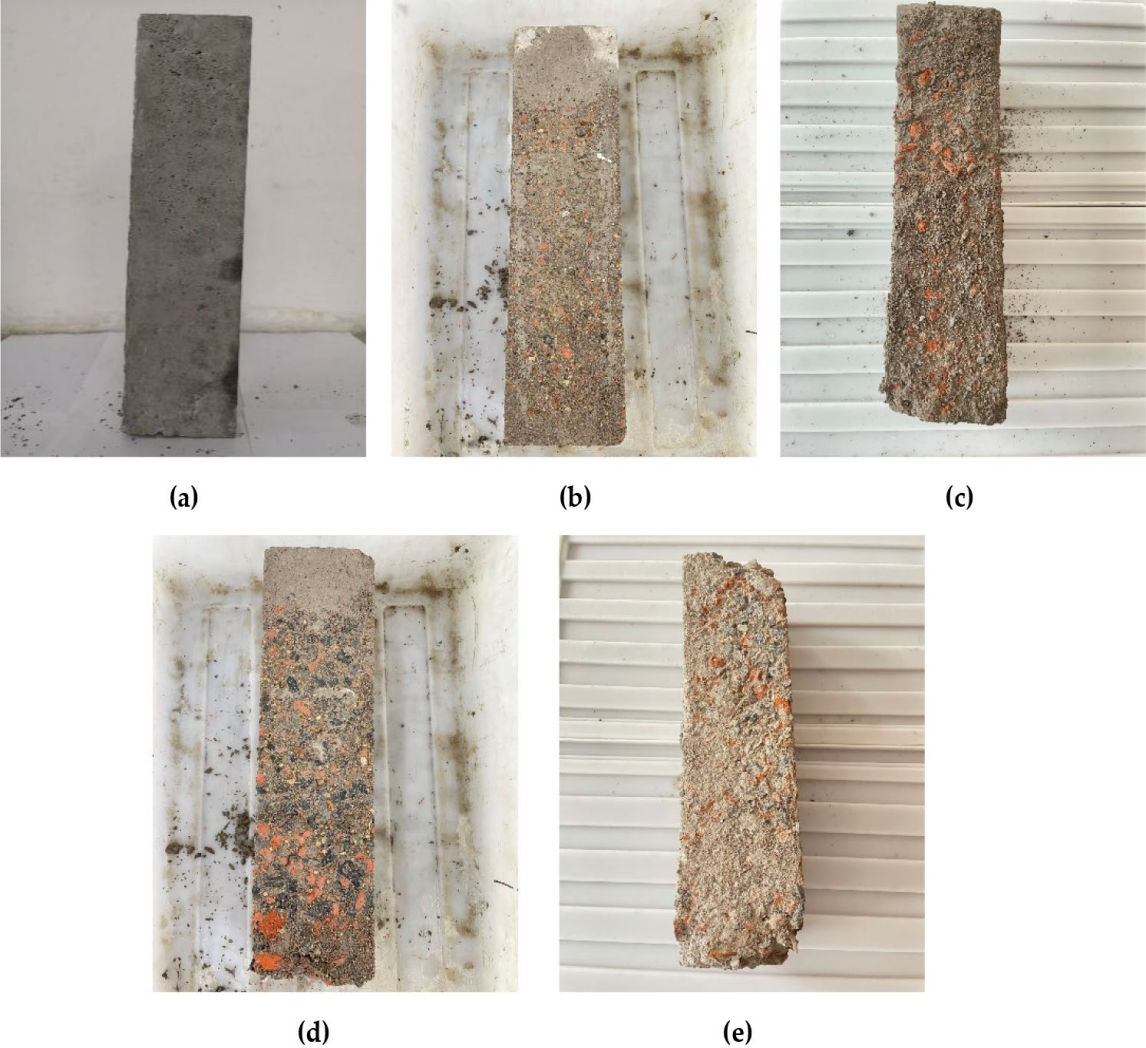
Specimen appearance after freezing and thawing: (**a**) Freeze–thaw 0 times; (**b**) Freeze–thaw 25 times; (**c**) Freeze–thaw 50 times; (**d**) Freeze–thaw 75 times; (**e**) Freeze–thaw 100 times.

The recycled concrete change in appearance were detected after every 25 times of freeze–thaw cycle as shown in Fig. 6. The mortar on the specimen surface gradually peeled off under the action of freeze–thaw cycles. With the increase in the replacement rate of recycled aggregate, the peeling phenomenon of the mortar surface was gradually aggravated. As a result, part of the red brick aggregate began to be exposed. With the increase in freeze–thaw cycles, the concrete specimens under various replacement rates had an aggravated frost damage trend. When the number of freeze–thaw cycles reached more than 50 times, the mortar on the surface of the concrete specimen began to fall off and accumulate at the bottom of the specimen, and the aggregate on the outer surface of the concrete specimen was exposed obviously. The structural integrity of the concrete specimens was damaged when the number of freeze–thaw reached 100 times, and part of the coarse aggregate began to fall off.

### Analysis of the damage mechanism

**Figure 7:**
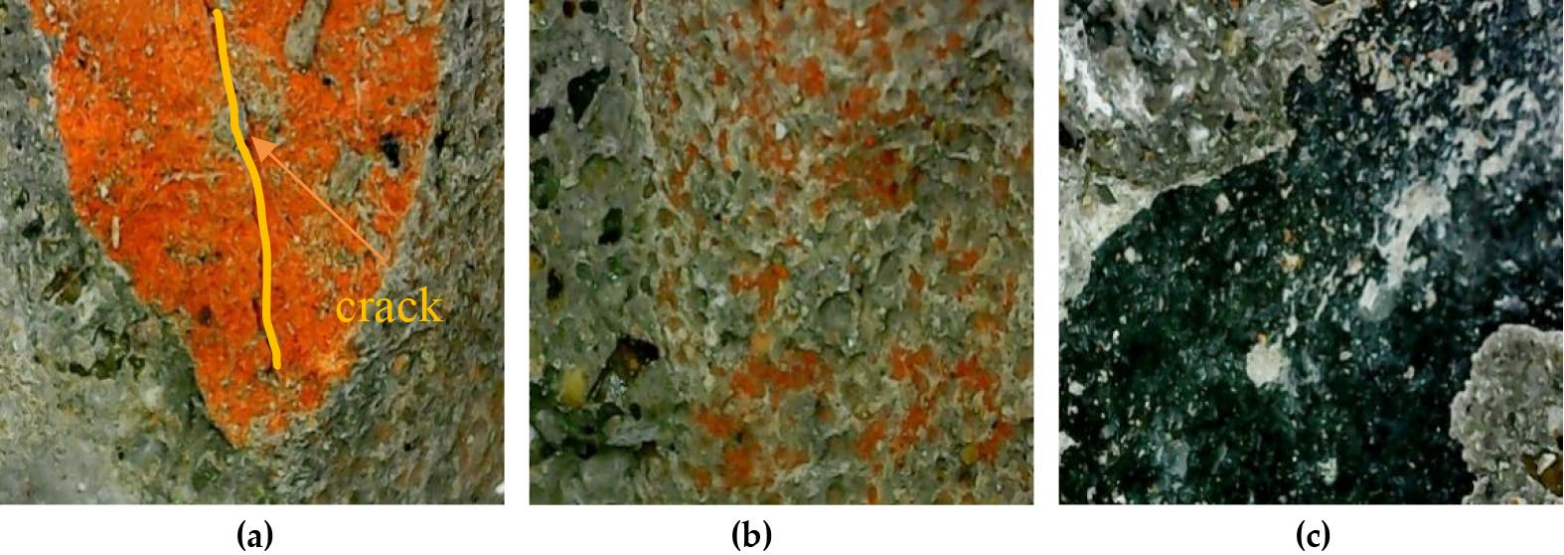
General microscope observation chart: (a) Red brick aggregates; (b) mortar; (c) Natural coarse aggregates.

**Figure 8:**
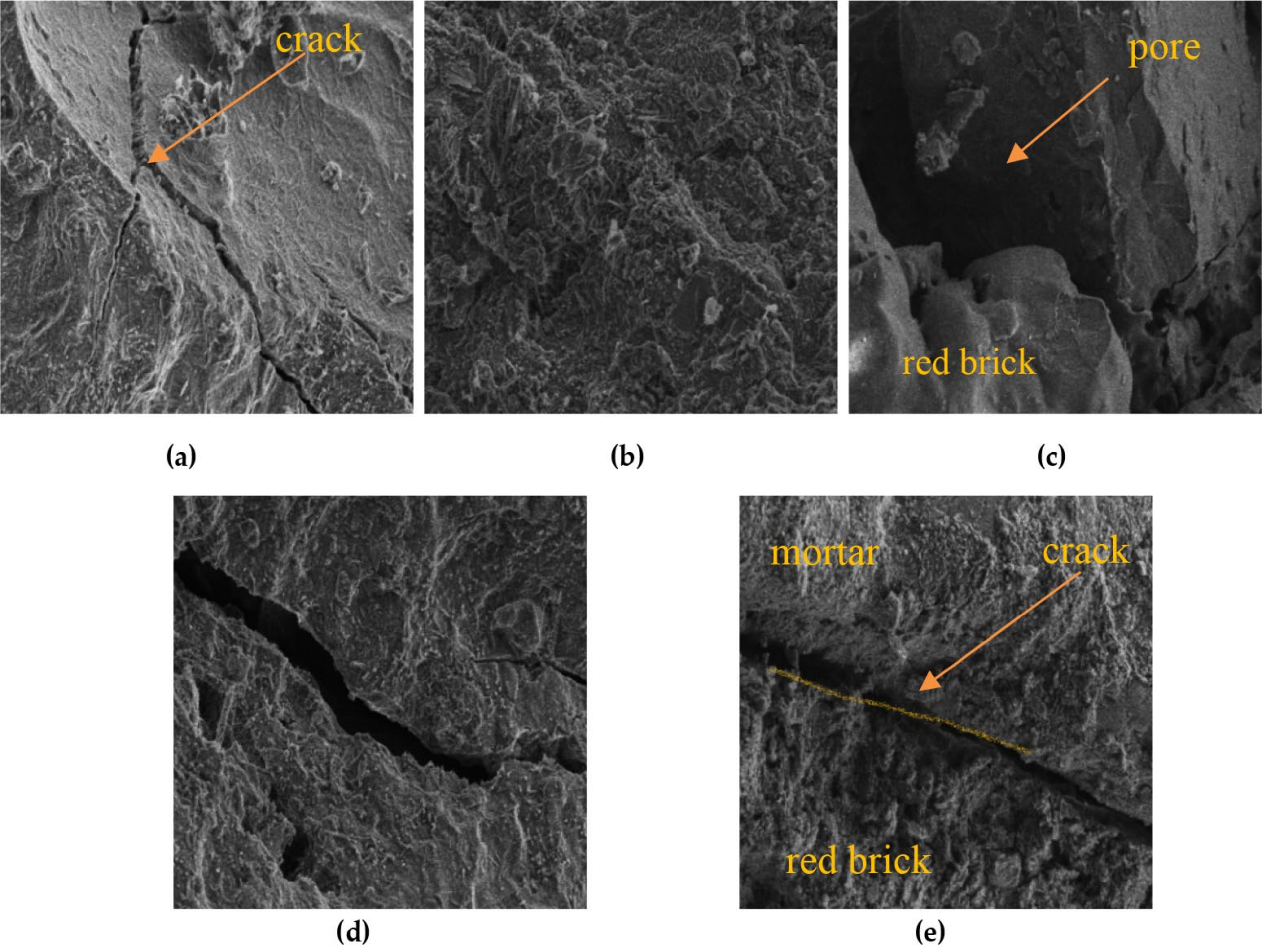
Electron microscope scanning SEM image: (**a**) Natural coarse aggregates; (**b**) Red brick aggregate surfaces; (**c**) Red brick aggregates; (**d**) Red brick aggregate cracks; (**e**) Red brick aggregate and mortar transition zone.

The damage sections of concrete specimens with 100 freeze–thaw cycles were randomly selected after flexural tests, and the red brick aggregate, natural coarse aggregate, and red brick aggregate wrapped with mortar were placed under digital microscope at 100 times magnification and scanned at 2000 times magnification under electron microscope, and the microscopic figures obtained through microscopic observation are shown in Figs. 7 and 8. It can be seen that cracks appeared on the surface of the stressed red brick aggregate after 100 times of freeze-thawing cycles under digital microscope observation (Fig. 7a). The adhesion between the red brick aggregate and mortar became poor during increasing freeze-thawing cycles, and part of the red brick aggregates began to fall off. The surface of the fallen mortar showed more small holes due to the freeze-thawing water absorption (Fig. 7b). However, the natural aggregate was less affected by the freeze–thaw cycle and external forces. The aggregate and mortar were wrapped closely linked (Fig. 7c), and no apparent cracks and aggregate shedding appeared under the digital microscope observation.

The natural aggregate surface is smoother in the scanned electron micrographs, as shown in Fig. 8a. The crack width of the aggregate from the damage section varies from about a few microns to more than ten microns. Compared with the natural coarse aggregate, the surface of red brick aggregate was rough with more pits and pores (Fig. 8b and c). The recycled brick aggregate has a loose internal structure with numerous pore cracks (Fig. 8d and Table 1). The crack width is mostly around 25 microns, leading to its crushing index, water absorption rate, and other parameters higher than natural aggregate. It can be explained that recycled brick aggregate absorbs more free water during the freeze–thaw cycles. The microscopic morphology of the transition zone at the interface between the coarse aggregate of the red brick and the new mortar is shown in Fig. 8e. The more apparent cracks were observed at the interface between the red brick and the mortar, and the crack's width varies from 15 to 30 µm. The water content inside the red brick aggregate increases with the increase of freeze–thaw cycles, and the structure becomes loose than that in the natural aggregate. The red brick aggregate influences the flexural strength in the case of undoped steel fiber, and the flexural strength decreases with the increase of freeze–thaw cycles. The specimen failure mode is more likely to occur when the aggregate is damaged.

### Quality loss rate

The test is carried out by the fast-freezing method, and the mass loss rate of the specimen is calculated as:

1 $$W=\frac{{M}_{0}-{M}_{n}}{{M}_{0}}\times 100\mathrm{\%}$$

where W is the rate of mass loss of concrete; M0 is the initial mass of concrete before freezing and thawing, expressed in grams (g); Mn is the mass of concrete after n freeze–thaw cycles, expressed in grams (g).

**Figure 9:**
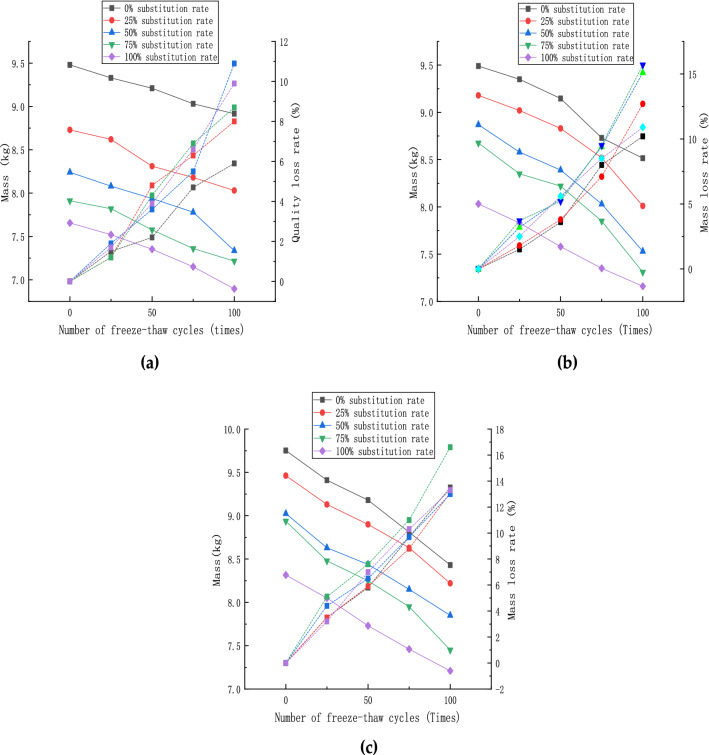
Specimens mass and mass loss rate: (**a**) steel fiber content 0%; (**b**) steel fiber content 1%; (**c**) steel fiber content 2%.

The quality tests of the specimens were conducted under saturated surface dry conditions. Figure 9 shows the specimens' measured masses and mass-loss rates with the increase of freeze–thaw cycles. Again, the masses in each group decreased to different degrees when comparing the specimens under each fiber dose. It can be found that with the increase in the recycled aggregate replacement rate, the mass loss rate of all groups increased. In addition, with the gradual increase of steel fiber admixture, the mass loss rate of the specimens gradually increased under the same recycled aggregate replacement rate, and the increase was above 10%. It was mainly concentrated in the specimens with 50 and 75% replacement rates. The specimens with 2% steel fiber and 75% coarse aggregate substitution rate had the most significant mass loss, which reached 17%. Compared with the normal concrete and the 100% recycled concrete specimens, the interface structure is more complicated, including the old mortar-new mortar, the old aggregate-new mortar, and the old aggregate-old mortar interface transition zone. There are still natural coarse and fine aggregates in the specimens under this substitution rate, which hinders the absorption of free water by the recycled aggregates. Furthermore, the steel fiber incorporation leads the compactness of the concrete to decrease, which makes the external structure of the specimen lose after freezing and causes some of the steel fibers to fall off so that the quality loss under these two replacement rates is prominent. With the increase of steel fiber, the mass loss rate of the specimens gradually increased, and in addition some of the steel fibers after the freezing and thawing started shedding and the mass-loss rate increased. It can be explained that the recycled aggregate itself has more internal micro-cracks and micro-voids. With increased pore thawing cycles, the external mortar surface started shedding and gradually exposed the internal recycled aggregate. As a result, its water absorption rate is much larger than the natural aggregate. Thus, the water absorption rate is much larger than that of natural aggregates, making the quality loss rate of the specimen decrease.

### Relative dynamic modulus

The relative dynamic elastic modulus is usually used to express the degree of damage inside the concrete. The fundamental transverse frequency of the specimen is determined by the resonance method. The fundamental transverse frequency is determined every 25 times of freezing and thawing. The relative dynamic elastic modulus is calculated using the following equation:

2 $${D}_{n}=\frac{{f}_{n}^{2}}{{f}_{0}^{2}}\times 100\%$$

where D_n_ is the relative dynamic elastic modulus of the specimen, %; f0 is the initial transverse fundamental frequency of the specimen, Hz; f_n_ is the fundamental transverse frequency of the specimen after n freeze–thaw cycles, Hz.

**Figure 10:**
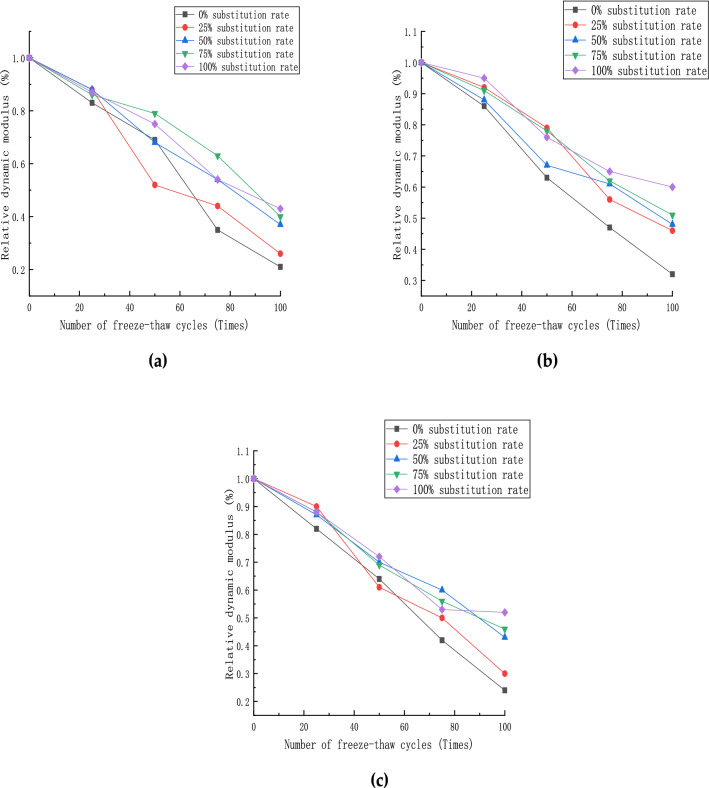
Relative dynamic modulus of specimens: (**a**) steel fiber content 0%; (**b**) steel fiber content 1%; (**c**) steel fiber content 2%.

The test specimens' relative dynamic elastic modulus data after the freeze–thaw cycle is completed, as shown in Fig. 10. It can be seen from the figure that with the increase in the number of freeze–thaw cycles, the relative dynamic elastic modulus of each group of specimens gradually decreases. The transverse analysis shows that each group of specimens' relative dynamic elastic modulus is better than the control group (0% of recycled aggregate). As the steel fiber content is 1%, the relative dynamic elastic modulus of the specimens performed up to 28% higher than the control group. The relative dynamic elastic modulus of the specimens with different steel fiber contents has rebounded when the ratio of coarse aggregate replacement is 100%, indicating that the concrete mortar surface has been completely peeled off in this case. Partial coarse and fine aggregates have also fallen off. After that, the damage process from the surface to the core concrete caused by the freeze–thaw cycle is gradually slowed down, and its internal structure stabilize. When the content of steel fiber is 2%, the relative dynamic elastic modulus of the specimen under the replacement rate of recycled aggregate is lower than that when the content is 1%, which indicates that the content of steel fiber is too much in this state. The internal structure of the specimen is more chaotic with the increase in freezing and thawing cycles.

### Flexural strength of recycled concrete

The prismatic specimen conversion coefficient is 0.85, and the flexural strength formula is:

3 $${f}_{t}=0.85\frac{Fl}{b{h}^{2}}$$

where f_t_ is flexural strength of concrete, MPa; F is damage load of concrete specimens, KN; *l* is span between supports, mm; b is width of concrete specimen section, mm; h is height of concrete specimen section, mm.

**Figure 11:**
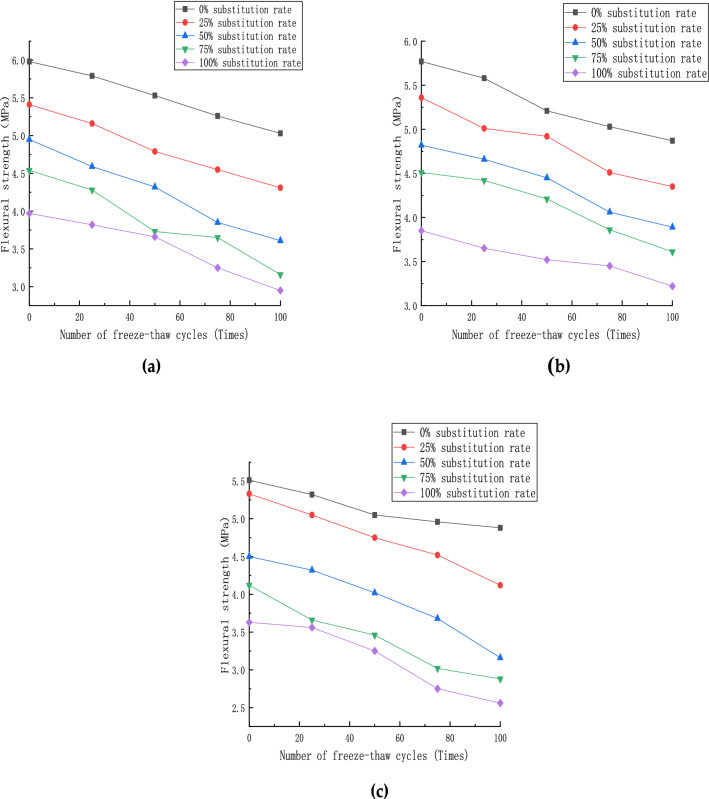
Flexural strength of specimen: (**a**) steel fiber content 0%; (**b**) steel fiber content 1%; (**c**) steel fiber content 2%.

The flexural strength of each specimen after the freeze–thaw cycle is completed is shown in Fig. 11. With the increase in the number of freeze–thaw cycles, the flexural strength of the specimen under each fiber content gradually decreases. When the red brick content is greater than or equal to 50%, the strength decreases gradually with the number of freeze–thaw increases.

For steel fiber content of 0% (Fig. 11a), the strength of ordinary concrete specimens decreases roughly linearly. In this case, the internal structure is seriously damaged, the red brick aggregate absorbs much free water, and the outer surface of the test piece falls off extremely, so the strength value changes drastically at this time.

For steel fiber content of 1% (Fig. 11b), the combination of steel fiber and concrete coarse and fine aggregates is relatively low. Thus, its flexural strength value reaches the maximum under the same conditions.

For steel fiber content of 2% (Fig. 11c), and the content of red brick reaches 50%, the free water content of the specimen is high due to the high water absorption rate of red brick aggregate. Hence, its flexural strength value fluctuates in a small range during the flexural test.

## Computational model analysis

### Quality loss rate calculation model

The change curve of the mass loss rate W and the number of freeze–thaw cycles n shows that the mass-loss rate M varies with the number of freeze–thaw cycles n in a roughly positive relationship, i.e.:

4 $$\mathrm{W}=\mathrm{an}+\mathrm{b}$$

Fitting analysis was performed by software and the values of slope a and intercept b were fitted by the least-squares method, as shown in Table 3.

**Table 3:** Results of γ-value parameter analysis (mass loss rate fitting).

γ	a	b	Steel fiber content (θ)	R^2^
0	0.06	− 0.14	0	0.98
25	0.08	− 0.16		0.95
50	0.10	− 0.70		0.99
75	0.09	− 0.40		0.94
100	0.10	0.10		0.93
0	0.11	− 0.72	1	0.94
25	0.12	− 1.06		0.94
50	0.15	− 0.66		0.97
75	0.15	− 0.62		0.93
100	0.11	0.11		0.97
0	0.13	− 0.14	2	0.99
25	0.13	− 0.04		0.99
50	0.13	0.46		0.97
75	0.16	0.24		0.98
100	0.13	0.13		0.99

Table 3 shows that a and b vary with the recycled aggregate substitution rate γ and steel fiber dose θ under different recycled aggregate substitution rate conditions and steel fiber dose. Thus, a and b are fitted as a quadratic function with the substitution rate γ, i.e., as shown in Eq. 5.

5 $$\begin{gathered} {\text{a}} = {\text{A}{\upgamma }}^{2} + {\text{B}{\upgamma }} + {\text{C}} \hfill \\ {\text{b}} = {\text{A}{\upgamma }}^{2} + {\text{B}{\upgamma}} + {\text{C}} \hfill \\ \end{gathered}$$

Therefore, the steel fiber content θ is presented by regression analysis, and the least-squares method is used to fit the values of A, B, and C in Eq. 5, respectively, as shown in Table 4.

**Table 4:** Results of θ-value parameter analysis (mass loss rate fitting).

	A	B	C	Steel fiber content (θ)	R^2^
a	− 0.06	0.09	0.06	0	0.95
b	2.15	− 2.05	− 0.04		0.92
a	− 0.15	0.16	0.10	1	0.99
b	2.03	− 1.19	− 0.76		0.96
a	− 0.03	0.05	0.13	2	0.99
b	− 1.30	1.63	− 0.20		0.92

The parameters A, B, and C are values related to the steel fiber content θ. The relationship between A, B, and C and the fiber content θ is fitted by the least squares method as Eq. 6.

6 $$\begin{gathered} {\text{a}}: \hfill\\ {\text{ A}} = 1050{\uptheta }^{2} - 19.5{\uptheta } - 0.06 \hfill\\ {\text{ B}} = - 900{\uptheta }^{2} + 16{\uptheta } + 0.09 \hfill\\ {\text{ C}} = - 50{\uptheta }^{2} + 4.5{\uptheta } + 0.06{ } \hfill \\ {\text{b}}: \hfill\\ {\text{ A}} = - 16050{\uptheta }^{2} - 148.5{\uptheta } + 2.15 \hfill\\ {\text{ B}} = 9800{\uptheta }^{2} - 12{\uptheta } - 2.05\hfill\\ {\text{ C}} = 6400{\uptheta }^{2} - 136{\uptheta } - 0.04 \hfill \\ \end{gathered}$$

Equations 5 and 6 are substituted into Eq. 4 to obtain the relationship between the mass-loss rate W and the steel fiber content θ, the substitution rate γ, and the number of cycles n.

7 $$\begin{gathered} {\text{W}} = \left[ {\left( {1050{{\uptheta }}^{2} - 19.5{{\uptheta }} - 0.06} \right){{\text{r}\upgamma }}^{2} + \left( { - 900{{\uptheta }}^{2} + 16{{\uptheta }} + 0.09} \right){{\upgamma }}} \right]{\text{n}} \hfill \\ \quad\quad\; \left. { + \left( { - 50{{\uptheta }}^{2} + 4.5{{\uptheta }} + 0.06} \right)} \right]{\text{n}} \hfill \\ \quad\quad\; + \left[ {\left( { - 16050{{\uptheta }}^{2} - 148.5{{\uptheta }} + 2.15} \right){{\upgamma }}^{2} + \left( {9800{{\theta }}^{2} - 12{{\uptheta }} - 2.05} \right){{\upgamma }}} \right. \hfill \\ \quad\quad\; + \left. { + \left( {6400{{\uptheta }}^{2} - 136{{\uptheta }} - 0.04} \right)} \right] \hfill \\ \end{gathered}$$

**Figure 12:**
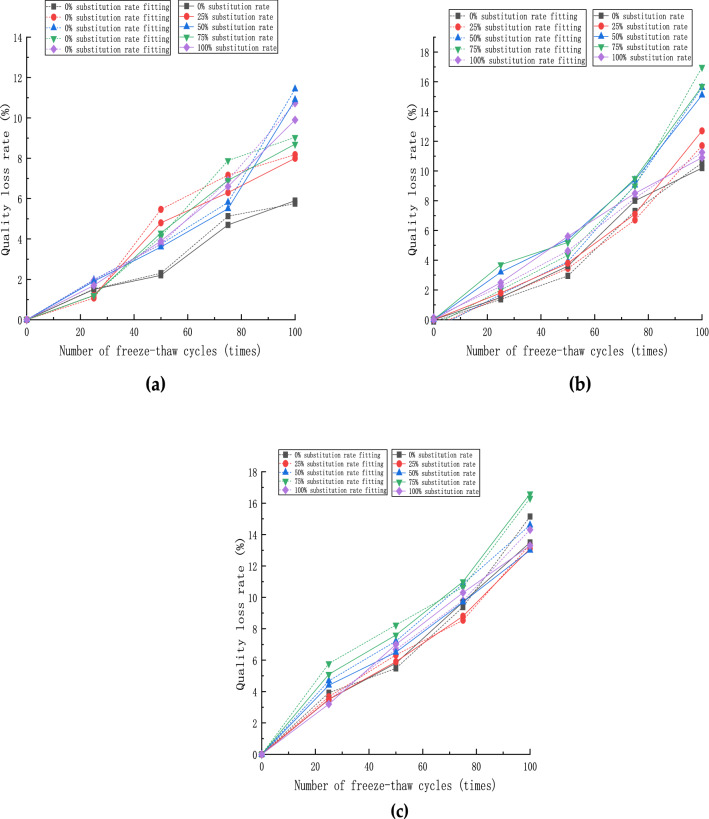
Mass loss rate fitting curve: (**a**) steel fiber content 0%; (**b**) steel fiber content 1%; (**c**) steel fiber content 2%.

The fitted curve of mass loss rate under each doping amount is shown in Fig. 12. As can be seen from the figure, when the steel fiber doping amount is 0%, the error between the test value and the fitted value is the largest, 7.8%, in the group of specimens with a 50% substitution rate at the number of freeze–thaw times of 100. On the other hand, the error of the remaining groups is within 5%, which is within a reasonable range. The slope of the fitted curve is similar to the experimental curve, so the fitted value of the calculated model (7) fits in the range of 0–100% substitution rate of recycled aggregate and 0–2% steel fiber doping. Thus, the presented numerical model can effectively predict the actual mass loss rate value in the above parameter range.

### Calculation model of relative dynamic elastic modulus

The observation of the variation curves of the relative dynamic mode Dn and the number of freeze-thaw cycles n shows that the relative dynamic mode Dn is positively correlated with the number of freeze-thaw cycles n.

8 $$\mathrm{Dn}=\mathrm{an}+\mathrm{b}$$

Fitting analysis was performed by software, and the least-squares method fitted the values of a and b, as shown in Table 5.

**Table 5:** Results of γ-value parameter analysis (relative dynamic elastic modulus fitting).

γ	a	b	Steel fiber content (θ)	R^2^
0	− 0.01	1.03	0	0.96
25	− 0.01	1.00		0.97
50	− 0.01	1.01		0.90
75	− 0.01	1.02		0.93
100	− 0.01	− 0.01		0.93
0	− 0.01	1.01	1	0.92
25	− 0.01	1.03		0.99
50	− 0.01	0.99		0.98
75	− 0.01	1.02		0.95
100	0.00	0.00		0.97
0	− 0.01	1.01	2	0.92
25	− 0.01	1.02		0.97
50	− 0.01	1.00		0.98
75	− 0.01	1.00		0.99
100	− 0.01	− 0.01		0.99

Table 5 shows that a and b vary with the recycled aggregate substitution rate γ and steel fiber dose θ under different recycled aggregate substitution rates and steel fiber dose conditions. Thus, a and b are fitted as a quadratic function with the substitution rate γ, i.e., as shown in Eq. 9.

9 $$\begin{gathered} {\text{a}} = {\text{A}{\upgamma}}^{2} + {\text{B}{\upgamma}} + {\text{C}} \hfill \\ {\text{b}} = {\text{A}{\upgamma}}^{2} + {\text{B}{\upgamma}} + {\text{C}} \hfill \\ \end{gathered}$$

Therefore, the steel fiber doping θ is presented by using the regression analysis. The values of A, B, and C in Eq. 9 are fitted using the least-squares method, respectively, as shown in Table 6.where the parameters A, B, and C are the values related to the steel fiber doping θ. The relationship between A, B, and C with fiber doping θ is fitted by least squares method as Eq. 10, respectively.

10 $$\begin{gathered} {\text{a:}} \hfill\\ \text{A} = - 200{\uptheta }^{2} + 4{\uptheta } \hfill\\ {\text{B}} = 100{\uptheta }^{2} - 2{\uptheta }\hfill\\ {\text{C}} = - 0.01 \hfill \\ {\text{b}}: \hfill\\ {\text{A}} = - {\uptheta } - 2.29 \hfill\\ {\text{ B}} = - 200{\uptheta }^{2} + 5{\uptheta } + 1.46\hfill\\ {\text{ C}} = 50{\uptheta }^{2} - 1.5{\uptheta } + 0.94 \hfill \\ \end{gathered}$$

**Table 6:** Results of θ-value parameter analysis (relative dynamic elastic modulus fitting).

	A	B	C	Steel fiber content (θ)	R^2^
a	0.00	0.00	− 0.01	0	0.92
b	− 2.29	1.46	0.94		0.92
a	0.02	− 0.01	− 0.01	1	0.99
b	− 2.30	1.49	0.93		0.93
a	0.00	0.00	− 0.01	2	0.96
b	− 2.31	1.48	0.93		0.92

Equations 9 and 10 are substituted into Eq. 8 to calculate the relative dynamic elastic modulus Dn related to the fiber doping θ, substitution rate γ, and freezing-thawing cycles n.

11 $$\begin{gathered} {\text{Dn}} = \left[ {\left( { - 200{{\uptheta }}^{2} + 4{{\uptheta }}} \right){{\upgamma }}^{2} + \left( {100{{\uptheta }}^{2} - 2{{\uptheta }}} \right){{\upgamma }} - 0.01} \right]{\text{n}} \hfill \\ \quad\quad\;\; + \left[ {\left( { - {{\uptheta }} - 2.29} \right){{\upgamma }}^{2} + \left( { - 200{{\uptheta }}^{2} + 5{{\uptheta }} + 1.46} \right){{\upgamma }}} \right. \hfill \\ \quad\quad\;\; \left. { + \left( {50{{\uptheta }}^{2} - 1.5{{\uptheta }} + 0.94} \right)} \right] \hfill \\ \end{gathered}$$

**Figure 13:**
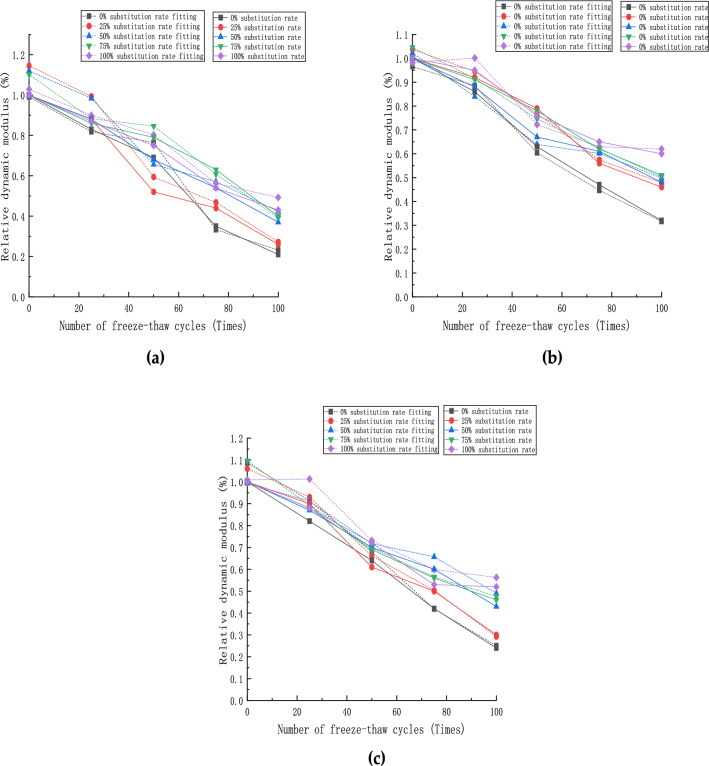
Relative dynamic elastic modulus fitting curve: (**a**) steel fiber content 0%; (**b**) steel fiber content 1%; (**c**) steel fiber content 2%.

The fitted curves of the relative dynamic modulus for each doping amount are shown in Fig. 13. It can be seen that the fitted curves calculated according to the calculation model (11) simulate the actual loss of relative dynamic modulus of concrete specimens well. Moreover, it can predict the loss of relative dynamic modulus of concrete specimens by changing recycled aggregate replacement rate and steel fiber doping amount. The slope of the fitted curves and the nodal values were in good agreement with the actual values in the range of 0–100% recycled aggregate substitution and 0–2% steel fiber content, and the trend of the relative dynamic modulus of recycled concrete specimens with different percent content could be predicted in the above range.

### Flexural strength calculation model

The observation of the variation curves of flexural strength ft and the number of freeze-thaw cycles n shows that the relative flexural strength ft is positively correlated with the number of freeze-thaw cycles n, i.e:

12 $$\mathrm{ft}=\mathrm{an}+\mathrm{b}$$

Fitting analysis was performed by software, and the least-squares method fitted the values of a and b, as shown in Table 7.

**Table 7:** Results of γ-value parameter analysis (Flexural strength fitting).

γ	a	b	Steel fiber content (θ)	R^2^
0	0.00	1.00	0	0.99
25	0.00	1.00		0.98
50	0.00	1.00		0.95
75	0.00	1.00		0.99
100	0.00	0.00		0.99
0	0.00	1.00	1	0.99
25	0.00	1.00		0.99
50	0.00	1.01		0.98
75	0.00	1.02		0.99
100	0.00	0.00		0.99
0	0.00	0.99	2	0.99
25	0.00	1.00		0.99
50	0.00	1.02		0.99
75	0.00	0.98		0.99
100	0.00	0.00		0.99

Table 7 shows that under different replacement rates of recycled aggregate and steel fiber content, a and b increase with the replacement rate of recycled aggregate γ and steel fiber content θ variation. Thus, a and b are calculated by performing quadratic function fitting related to the substitution rate γ, as shown in Eq. 13.

13 $$\begin{gathered} {\text{a}} = {\text{A}{\upgamma}}^{2} + {\text{B}{\upgamma}} + {\text{C}} \hfill \\ {\text{b}} = {\text{A}{\upgamma}}^{2} + {\text{B}{\upgamma}} + {\text{C}} \hfill \\ \end{gathered}$$

Therefore, the steel fiber doping θ is presented using regression analysis. The A, B, and C values in Eq. 13 are fitted using the least-squares method, respectively, as shown in Table 8.

**Table 8:** Results of θ-value parameter analysis (Flexural strength fitting).

	A	B	C	Steel fiber content (θ)	R^2^
a	0.00	0.00	0.00	0	1.00
b	− 2.29	1.49	0.91		0.98
a	0.00	0.00	0.00	1	0.99
b	0.00	0.02	0.91		0.99
a	0.00	0.00	0.00	2	0.99
b	− 2.33	1.53	0.91		0.99

The parameters A, B, and C are the values related to the steel fiber doping θ, the relationship between A, B, and C with steel fiber doping θ was fitted by the least-squares method as Eq. 14, respectively.

14 $$\begin{gathered} {\text{a:}}\hfill \\ {\text{A}} = 0\; \hfill \\{\text{B}} = 0\; \hfill \\ {\text{C}} = 0 \hfill \\ {\text{b}}: \hfill \\ {\text{A}} = - 23100{\uptheta }^{2} + 460{\uptheta } - 2.29 \hfill \\ {\text{ B}} = 14900{\uptheta }^{2} - 296{\uptheta } + 1.49 \hfill \\ {\text{ C}} = 0.91 \hfill \\ \end{gathered}$$

Equations 13 and 14 are substituted into Eq. 12 to calculate the flexural strength ft related to the fiber doping θ, substitution rate γ, and freezing cycles n.

15 $$\begin{gathered} {\text{f}}_{{\text{t}}} = \left[ {\left( { - 23100{{\uptheta }}^{2} + 460{{\uptheta }} - 2.29} \right){{\gamma }}^{2} } \right]{\text{n}} \hfill \\ \quad\quad + \left( {14900{{\theta }}^{2} - 296{{\uptheta }} + 1.49} \right){{\upgamma }} + 0.91 \hfill \\ \end{gathered}$$

As shown by Eq. 15, the relative flexural strength degradation is less related to the number of freeze–thaw cycles and more related to the fiber doping and substitution rate, which is consistent with what the graphs show.

**Figure 14:**
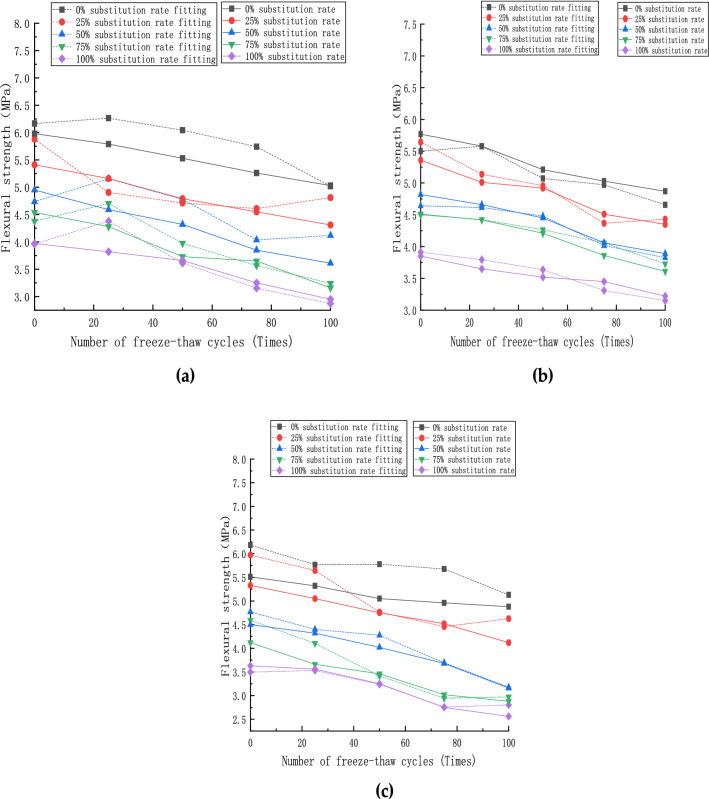
Flexural strength fitting curve: (**a**) steel fiber content 0%; (**b**) steel fiber content 1%; (**c**) steel fiber content 2%.

The fitted curves of relative dynamic modulus under each doping amount are shown in Fig. 14. Combined with the calculated model (15), it can be seen that the degradation of flexural strength has little relationship with the number of freeze-thaw cycles and is more related to the steel fiber doping amount and recycled aggregate replacement rate. As shown in Fig. 14a, the fitted value deviates partially from the test value in the case of a low recycled aggregate replacement rate. The maximum deviation value is 8.6%, and the change of error value under this dose is mainly affected by the recycled aggregate replacement rate. The test value has some errors, but it is within a reasonable range. The experimental and test curves under the doping amount fit relatively well. In the above range of recycled aggregate replacement rate and steel fiber dosing, the fitted curves calculated according to the calculation model (15) can better simulate the degradation process of flexural strength of concrete specimens. It can better predict the degradation of flexural strength of concrete specimens with the variation of recycled aggregate replacement rate and steel fiber dosing.

### Relevance analysis

The gray correlation model is a multi-factor statistical analysis method based on the sample data of each system. It uses gray correlation degree to describe the relationship between factors’ strength, size, and order. The correlation between two systems is judged according to the size of the correlation degree, and the larger the correlation degree is, the higher the correlation between the two systems, and the closer the speed, direction, and size change of the two systems. The specific steps of the method are as follows:

1. This test is carried out based on the mass loss rate, relative dynamic elastic modulus, and flexural strength to determine the reference sequence X0(K) = {X0(1), X0(2), X0(3),…X0(n)}, where n means The comparison sequence Xi(K) = { Xi(1), Xi(2), Xi(3),…Xi(n)}, i represents the change in the substitution rate of steel fibers and recycled aggregates and the change in the number of freeze–thaw cycles, n represents the type of test performed
2. The mean value method is used to dimensionless process each sequence, and the formula is as follows:
  $${\mathrm{X}}_{\mathrm{i}}^{\mathrm{^{\prime}}}\left(\mathrm{k}\right)=\frac{{\mathrm{X}}_{\mathrm{i}}(\mathrm{k})}{\frac{1}{\mathrm{n}}\sum_{\mathrm{k}=1}^{\mathrm{n}}{\mathrm{X}}_{\mathrm{i}}(\mathrm{k})}$$
3. Determine the absolute difference series with the following equation:
  $${\Delta }_{\mathrm{i}}\left(\mathrm{k}\right)=\left|{\mathrm{X}}_{0}^{\mathrm{^{\prime}}}\left(\mathrm{k}\right)-{\mathrm{X}}_{\mathrm{i}}^{\mathrm{^{\prime}}}\left(\mathrm{k}\right)\right|$$
4. Calculate the maximum difference M between the two levels and the minimum difference m between the two levels with the following equation:
  $${\text{M}} = \max_{{\text{i}}} \max_{{\text{k}}} \Delta_{{\text{i}}} (k),\;\;{\text{m}} = \min_{{\text{i}}} \min_{{\text{k}}} \Delta_{{\text{i}}} ({\text{k}})$$
5. Calculate the correlation coefficient with the following formula:
  $$\gamma_{{0{\text{i}}}} ({\text{k}}) = \frac{{{\text{m}} + \varepsilon {\text{M}}}}{{\Delta_{{\text{i}}} ({\text{k}}) + \varepsilon {\text{M}}}}$$
  where ε is the resolution factor, generally taken as 0.5.
6. Calculate the gray correlation degree with the following equation.
  $$\gamma_{{0{\text{i}}}} = \frac{1}{{\text{n}}}\sum\limits_{{{\text{k}} = 1}}^{{\text{n}}} {\gamma_{{0{\text{i}}}} } ({\text{k}})$$

The grey correlation model was performed three times, and the resulting correlation values are shown in Table 9.

**Table 9:** Numerical results of correlation analysis.

	γ1	γ2	γ3
Quality loss rate	0.654969	0.859759	0.651996
Relative modulus of elasticity	0.580477	0.548062	0.631075
Flexural strength	0.557419	0.552388	0.540953

According to the correlation degree data, under the comprehensive action of steel fiber content, freeze–thaw cycle times, and recycled aggregate replacement rate, the correlation degree of each parameter index changes significantly. Among them, the correlation degree between mass-loss rate and freeze-thaw cycle times is the largest, 0.859759; The second is the content of steel fiber, which is 0.654969; The correlation degree with substitution rate is the smallest, which is 0.651996. The correlation between relative dynamic modulus and substitution rate is the largest, which is 0.631075; The second is the content of steel fiber, and the correlation degree is 0.580477; The correlation degree with the number of freeze-thaw cycles is the smallest, which is 0.548062. The correlation degree between flexural strength and steel fiber content is the largest, 0.557419; The second is the number of freeze-thaw cycles, and the correlation degree is 0.552388; The correlation degree with substitution rate was the smallest, 0.540953.

## Conclusions

The mechanical test process of steel fiber recycled brick aggregate concrete and the changes in mechanical properties and durability performance are similar to those of ordinary concrete under the same conditions. However, the degree of deterioration of its mechanical properties and durability performance after freeze-thaw cycles and the damage mechanism are influenced by the replacement rate of red brick aggregate and the amount of steel fiber admixture.

1. The damage pattern of steel fiber recycled brick aggregate specimens after stress is similar to that of ordinary concrete. However, the brittleness of the specimens is increased after mixing with red brick aggregate, and mixing with steel fiber can effectively alleviate the time of specimen damage and improve the ductility of the specimens. The average relative dynamic modulus of concrete specimens was increased by 19.25%, and the average flexural strength was increased by 29% when the steel fiber admixture was 1%.
2. The damaged section of the specimen after freezing and stressing was scanned by electron microscope. It was found that the red brick aggregate with a rough surface and more pores was more likely to absorb more free water after freezing, which in turn led to its mass loss rate slowing down instead with the increase of the number of freeze–thaw cycles.
3. When the steel fiber admixture is 2%, with the increase in recycled aggregate replacement rate, the concrete absorbs more free water during mixing and freeze–thaw cycles, making its mechanical property indexes show a decreasing trend. Combined with the comprehensive analysis of the state of recycled aggregate under the electron microscope, it can be seen that in the case of higher steel fiber admixture and recycled aggregate replacement rate greater than 50%, the free water content in the specimen is minor. The concrete compactness of the specimen is reduced, resulting in a decrease in its mechanical property index. Therefore, this kind of concrete in the practical application process should be added to the appropriate ratio of water-reducing agents or improve the quality of water in the ratio.
4. The calculated model derived from the software analysis fits well with the test curve and can predict the degradation of the specimen's strength index and durability index under freeze–thaw conditions.
5. The gray correlation analysis can analyze the degree of influence of each test index by different variables in different test environments. The degree of influence of each doping index changes on the test results. Therefore, different environments need to be considered in practical application to make appropriate adjustments to the mix ratio of concrete structures.

## Data Availability

All data generated or analysed during this study are included in this published article.
